# Antimicrobial Resistance and Molecular Epidemiology of *Staphylococcus aureus* Causing Bloodstream Infections at Ruijin Hospital in Shanghai from 2013 to 2018

**DOI:** 10.1038/s41598-020-63248-5

**Published:** 2020-04-07

**Authors:** Feifei Gu, Weiping He, Shuzhen Xiao, Su Wang, Xinxin Li, Qian Zeng, Yuxing Ni, Lizhong Han

**Affiliations:** 1 Department of Clinical Microbiology, Ruijin Hospital, Shanghai Jiao Tong University School of Medicine, Shanghai, China; 2 Department of Laboratory Medicine, Huadong Hospital Affiliated to Fudan University, Shanghai, China

**Keywords:** Bacterial infection, Clinical microbiology

## Abstract

*Staphylococcus aureus* or methicillin-resistant *Staphylococcus aureus* (MRSA) is an important issue associated with significant morbidity and mortality and well known as a predominant pathogen causing bloodstream infection (BSIs) globally. To estimate the antibiotic resistance and molecular characteristics of *S. aureus* causing BSIs in Shanghai, 120 *S. aureus* isolates (20 isolates each year) from the patients with *S. aureus* BSIs from 2013 to 2018 were randomly selected and enrolled in this study. Fifty-three (44.2%) MRSA isolates were determined, and no isolate was found resistant to vancomycin, daptomycin, synercid, linezolid and ceftaroline. The toxin genes *tst*, *sec*, *seg* and *sei* were found more frequently among MRSA isolates compared with MSSA isolates (all *P* < 0.0001). Twenty-nine sequence types (STs) were identified, and ST5 (23.3%) was the most common ST, followed by ST398 (11.7%) and ST764 (10.0%). SCC*mec* II (73.6%) was the most frequent SCC*mec* type among MRSA isolates. The dominant clonal complexes (CCs) were CC5 (ST5, ST764, ST965 and ST3066; 36.7%) and the livestock-associated clone CC398 (ST398, 11.7%). MRSA-CC5 was the predominant CC among MRSA isolates (37/53, 69.8%), and CC5-II MRSA was found in 34 isolates accounting for 91.9% (34/37) among CC5 MRSA isolates. In addition, all 29 *tst*-positive MRSA isolates were CC5-MRSA as well. Our study provided the properties and genotypes of *S. aureus* causing BSIs at Ruijin Hospital in Shanghai from 2013 to 2018, and might suggest of value clues for the further study insights into pathogenic mechanisms intrinsically referring to the development of human-adapted *S. aureus* clones and their diffusions.

## Introduction

Bloodstream infections (BSIs) is a severe infection with high incidence and lethality all over the world, and it always prolonged hospital stay for a long period^1^. It has been reported to be one of the seven leading causes of death in North America and Europe^2^. *Staphylococcus aureus* is well known as one of the most important human pathogens across the world and is capable of causing a variety of infections in healthcare facilities and communities. Furthermore, *S. aureus* is one of the major and most fatal causes of bacteremia with an estimated mortality of 20%, and at least 50% of patients with *S. aureus* bacteremia (SAB) will develop complicated bacteremia^3^.

*S. aureus* is one of the most common causes of severe BSIs with high morbidity and mortality. Early mortality associated with SAB appears to have plateaued at approximately 20–30%^4^, and imposes a substantial burden on patients and healthcare systems. In the United States, the annual incidence of SAB is 4.3 to 38.2 per 100,000 person-years, and the 30-day all-cause mortality of SAB is 20% and has not changed since the 1990s^5^. In Ireland, *S. aureus* accounted for approximately 19–32% annually among BSIs and has been the second most common cause of BSIs consistently, ranking only second to *Escherichia coli* between 2006 and 2016 as reported^6^. Besides, *S. aureus* is also the second-most frequent pathogen found in neonatal bacteremia in the UK, resulting in significant neonatal morbidity and mortality that approximately 20–35%^7^.In China from 2012 to 2016, *S. aureus* accounted for 25.9% among BSIs in Jiangxi province, which is located in Southeastern China^8^. The European Antimicrobial Resistance Surveillance System including 27 countries revealed an increased burden of *S*. *aureus* BSIs by 2.3% per year in the 2000s and rising levels of *S*. *aureus* BSIs due to methicillin-susceptible *S*. *aureus* (MSSA)^9^. In Alberta, Canada, there were 299 cases of methicillin-resistant *S*. *aureus* (MRSA) BSIs in total from 2011 to 2013, equating to 3.95 cases per 100,000 population as reported^10^. The epidemiology of *S*. *aureus* among BSIs appears to be changing, and several surveillances worldwide reveal an increasing incidence of BSIs with *S*. *aureus*^11^.

*S. aureus* features among for its versatility in adaptability, ability to capture various resistance, virulence genes and genetic diversity. Thus, our study aimed to investigate the antimicrobial resistance and molecular characteristics of *S. aureus* causing BSIs in Shanghai, China from 2013 to 2018.

## Materials and Methods

### Study design and bacterial isolates

This study was performed at Ruijin Hospital, Shanghai Jiao Tong University School of Medicine, a tertiary teaching hospital with more than 3,000 beds serving patients from all over China. Because of the transferring of laboratory information system at Ruijin Hospital in 2015, the detail information of BSIs from January 2013 to July 2015 were missing. From August 2015 to December 2018, a total of 2760 patients with BSIs were determined at Ruijin Hospital, and 204 (7.4%) of them were caused by *S. aureus*.

All patients with BSIs in this study were determined by blood culture. Only the first positive blood cultures were reviewed and recorded. The initial species identification was performed using MALDI-TOF MS (bioMérieux, Marcy-l’Étoile, France). From January 2013 to December 2018, 243 *S. aureus* isolated from the patients with SAB were collected. One hundred and twenty *S. aureus* isolates were randomly selected (20 isolates each year) and enrolled in this study using the random number generation function in Microsoft Office Excel 2016 (Microsoft Corporation, Redmond, WA, USA). This study was approved by Ethics Committee of Ruijin Hospital affiliated to Shanghai Jiao Tong University School of Medicine (2019 LinLunShen No.146), and the Review Committee removed the need for informed consent for this retrospective study which focused on bacteria and did not involve patient interventions.

### Antimicrobial susceptibility testing

Antimicrobial susceptibility testing was performed manually according to the guidelines of Clinical and Laboratory Standards Institute issued in 2018 (CLSI 2018)^12^. Antibiotics tested were as following using broth microdilution method: penicillin(0.03,0.12–0.25,2,8 μg/ml), cefoxitin screen(4 μg/ml), oxacillin(0.25,1–2 μg/ml), erythromycin(0.5–4 μg/ml), clindamycin(0.25–0.5,2 μg/ml), azithromycin(2–4 μg/ml), gentamicin(4–8 μg/ml), levofloxacin(1–4 μg/ml), moxifloxacin(0.5–1,4 μg/ml), ciprofloxacin(1–2 μg/ml), cefazolin(8–16 μg/ml), rifampin(1–2 μg/ml), ampicillin(2–8 μg/ml), ampicillin/sulbactam(8/4–16/8 μg/ml), amoxicillin/K Clavulanate(4/2 μg/ml), tetracycline(4–8 μg/ml), chloramphenicol(8–16 μg/ml), trimethoprim/sulfamethoxazole(0.5/9.5–2/38 μg/ml), vancomycin(0.5–16 μg/ml), daptomycin(1,4 μg/ml), synercid(0.5–2 μg/ml), linezolid(2–4 μg/ml), ceftaroline(0.5–2 μg/ml). The antibiotics selected and their ranges were referred to MicroScan Pos Combo Panel Type 44 (Beckman Coulter, Inc. USA). *S. aureus* ATCC29213 strain was used as the quality control for the antimicrobial susceptibility testing.

### Toxin genes detection

A total of 13 significant toxin genes clinically including *lukS/F-PV*, *tst*, *eta*, *etb*, *sea*-*see* and *seg*-*sej* were detected on all 120 *S. aureus* isolates in this study by polymerase chain reaction (PCR) as described previously^13^. *lukS/F-PV* encodes Panton-Valentine leukocidin; *tst* encodes toxic shock syndrome toxin 1; *eta* and *etb* encodes exfoliative toxin A and B; *sea*-*see* and *seg*-*sej* encodes staphylococcal enterotoxins SEA-SEE and SEG-SEJ.

### Molecular typing

Multilocus sequence typing (MLST), *spa* typing and *agr* typing were performed on all 120 *S. aureus* isolates according to the guidelines on the websites (https://pubmlst.org/, http://spa.ridom.de/index.shtml) and other published documents and researches^13,14^. *mecA* detection was performed on all 120 *S. aureus* isolates as well to confirm the existence of MRSA. SCC*mec* types of MRSA were determined by the previous method as described^15^. The gene *blaZ*, which produces beta-lactamase and inactivates penicillin by hydrolyzing the beta-lactam ring, was detected on all 120 *S. aureus* isolates^16^.

### Statistical analysis

The chi-square or Fisher’s exact test was used for statistical analysis as appropriate, and a two-sided *P* value of <0.05 was considered for statistical significance. All statistical analysis in this study was conducted by the software package SAS 8.2 (SAS Institute Inc., Cary, NC, USA).

## Results

### Clinical data

From January 2013 to December 2018, the median age of patients with SAB in this study was 59 years (range: 7 months-97 years; interquartile range: 44–69 years), and the sex distribution (male/ female) was 67.5%/32.5%. The mortality of patients with SAB was 25.8%, while 15 (12.5%) patients were transferred with unknown outcomes.

The incidence of *S. aureus* accounting for BSIs was 7.4% at Ruijin Hospital in Shanghai from August 2015 to December 2018, for the system transferring in 2015 and data missing as described in materials and methods.

### Antimicrobial resistance

Fifty-three (44.2%) *S. aureus* isolates were confirmed as MRSA in this study, and all 53 MRSA isolates were *mecA*-positive. We did not discover any isolate resistant to vancomycin, daptomycin, synercid, linezolid and ceftaroline. No isolate was found showing a reduced vancomycin susceptibility or intermediate to vancomycin among the 120 *S. aureus* isolates in this study (supplementary information). The resistance rates of antibiotics tested for overall 120 *S. aureus* isolates from 2013 to 2018 were presented in Table 1. All 53 MRSA isolates detected in the study were resistant to penicillin, and 51 (51/67, 76.1%) MSSA isolates were resistant to penicillin. Sixty-eight (56.7%) *S. aureus* isolates including 17 MRSA and 51 MSSA isolates were detected *blaZ*-positive, and all 51 MSSA isolates resistant to penicillin were *blaZ*-positive as well. Forty-six (46/53, 86.8%) MRSA isolates were observed showing multi-drug resistance in this study.

**Table 1:** The antibiotic resistance rates of *Staphylococcus aureus* isolates causing bloodstream infections from 2013 to 2018.

Antibiotics	Resistance rate(%)								
	2013 (n = 20)	2014 (n = 20)	2015 (n = 20)	2016 (n = 20)	2017 (n = 20)	2018 (n = 20)	2013–2018		
							Total (n = 120)	MSSA (n = 67)	MRSA (n = 53)
Penicillin	100.0	95.0	80.0	80.0	90.0	75.0	86.7	76.1	100.0
Cefoxitin Screen	50.0	40.0	40.0	55.0	30.0	50.0	44.2	0.0	100.0
Oxacillin	50.0	40.0	40.0	55.0	30.0	50.0	44.2	0.0	100.0
Erythromycin	70.0	75.0	55.0	70.0	45.0	40.0	59.2	34.3	90.6
Clindamycin	45.0	55.0	35.0	60.0	25.0	15.0	39.2	17.9	66.0
Azithromycin	70.0	75.0	50.0	70.0	50.0	50.0	60.8	37.3	90.6
Gentamicin	40.0	40.0	10.0	40.0	35.0	15.0	30.0	10.4	54.7
Levofloxacin	45.0	45.0	35.0	55.0	30.0	35.0	40.8	9.0	81.1
Moxifloxacin	45.0	45.0	35.0	50.0	30.0	35.0	40.0	7.5	81.1
Ciprofloxacin	50.0	45.0	35.0	45.0	30.0	35.0	40.0	9.0	79.2
Cefazolin	45.0	40.0	25.0	50.0	25.0	30.0	35.8	0.0	81.1
Rifampin	5.0	0.0	5.0	0.0	0.0	5.0	2.5	0.0	5.7
Ampicillin	65.0	60.0	50.0	50.0	30.0	40.0	49.2	14.9	92.5
Ampicillin/Sulbactam	45.0	40.0	30.0	55.0	25.0	35.0	38.3	1.5	84.9
Amoxicillin/K Clavulanate	45.0	40.0	30.0	55.0	20.0	30.0	36.67	1.49	81.13
Tetracycline	50.0	45.0	30.0	35.0	25.0	30.0	35.8	9.0	69.8
Chloramphenicol	10.0	35.0	20.0	20.0	15.0	0.0	16.7	17.9	15.1
Trimethoprim/Sulfamethoxazole	10.0	5.0	0.0	0.0	5.0	5.0	4.2	0.0	9.4
Vancomycin	0	0	0	0	0	0	0	0	0
Daptomycin	0	0	0	0	0	0	0	0	0
Synercid	0	0	0	0	0	0	0	0	0
Linezolid	0	0	0	0	0	0	0	0	0
Ceftaroline	0	0	0	0	0	0	0	0	0

### Virulence factors

The toxin genes *etb* and *see* were not discovered among all *S. aureus* isolates in this study. The *seg* was found most frequently among the toxin genes screened, occurring in 60 isolates (50.0%) as presented in Table 2. The *tst*, *sec*, *seg* and *sei* were found more frequently among MRSA isolates compared with MSSA isolates (all *P* < 0.0001). However, *sed* was observed more frequently among MSSA isolates (*P* = 0.0254), and *sed* was detected only among eight MSSA isolates as shown in Table 2. Besides, *sej* was found only among four MSSA isolates, but there was no significant difference statistically between MSSA and MRSA isolates (*P* = 0.1946).

**Table 2:** Prevalence of toxin genes among *Staphylococcus aureus* isolates causing bloodstream infections from 2013 to 2018.

Toxin genes	Positive rate (%)			
	Total (n = 120) n (%)	MSSA (n = 67) n (%)	MRSA (n = 53) n (%)	*P* value
*lukS/F-PV*	1 (0.8)	0	1 (1.9)	0.4417
*tst*	32 (26.7)	3 (4.5)	29 (54.7)	<0.0001
*eta*	1 (0.8)	1 (1.5)	0	1.0000
*etb*	0	0	0	—
*sea*	18 (15.0)	7 (10.4)	11 (20.8)	0.1164
*seb*	5 (4.2)	1 (1.5)	4 (7.5)	0.2345
*sec*	33 (27.5)	4 (6.0)	29 (54.7)	<0.0001
*sed*	8 (6.7)	8 (11.9)	0	0.0254
*see*	0	0	0	—
*seg*	60 (50)	22 (32.8)	38 (71.7)	<0.0001
*seh*	55 (45.8)	26 (38.8)	29 (54.7)	0.0824
*sei*	57 (47.5)	20 (29.9)	37 (69.8)	<0.0001
*sej*	4 (3.3)	4 (6.0)	0	0.1946

### Molecular types

**Figure 1:**
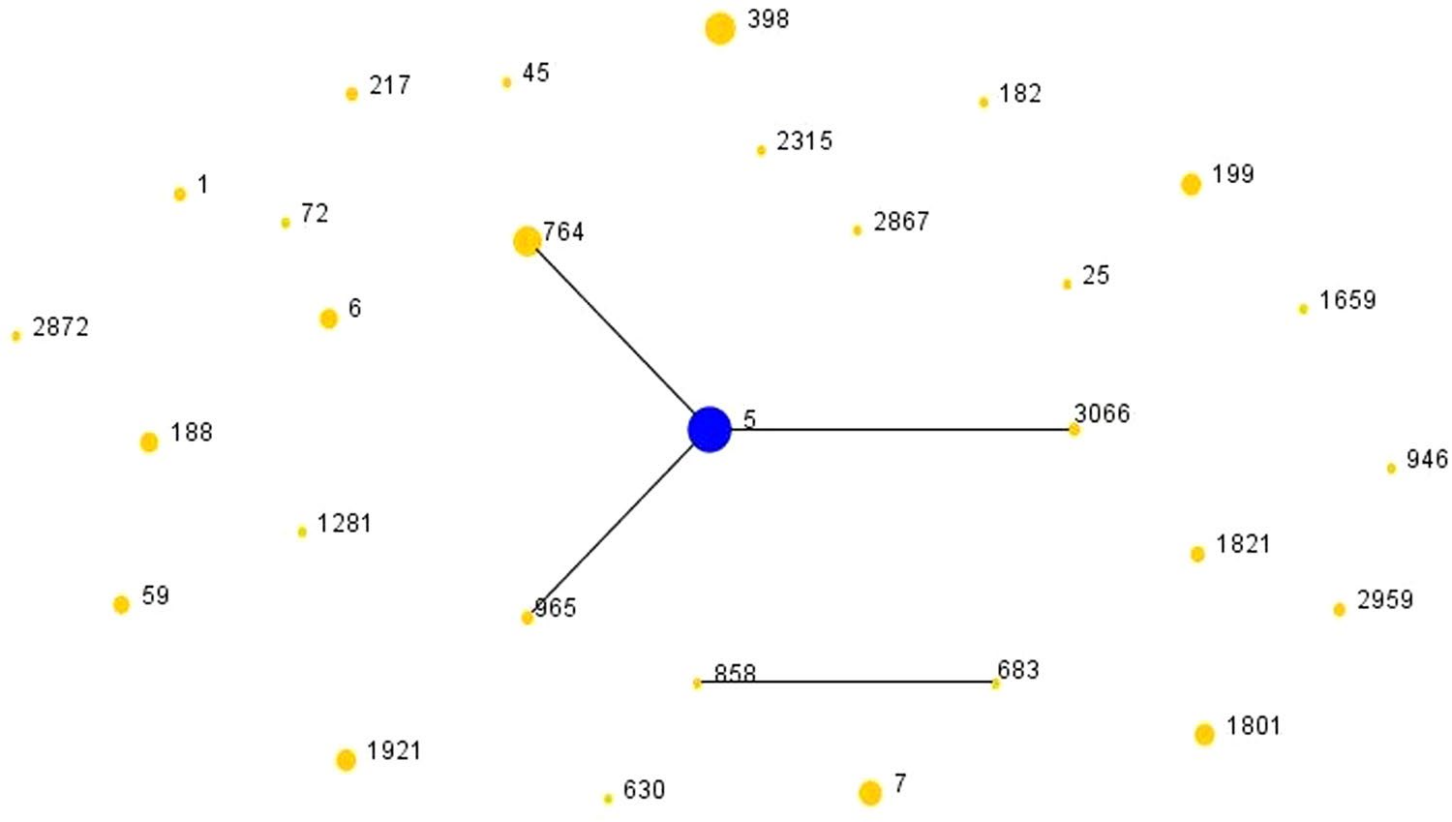
The diagram produced by eBURST with the stringent (default) group definition. Each number represents an MLST ST and the area of each circle indicates the prevalence of the ST in the MLST data of this study. MLST, multilocus sequence typing; ST, sequence type.

Twenty-nine sequence types (STs) were identified among all 120 *S. aureus* isolates as presented in Table 3. ST5 (28/120, 23.3%) was the most common ST, followed by ST398 (14/120, 11.7%) and ST764 (12/120, 10.0%); and t002 (12/120, 10.0%) was the most common *spa* type followed by t2460 (10/120, 8.3%) and t034 (7/120, 5.8%). One ST2959 MSSA isolate could not be *spa* typed (negative for certain PCR). Among 53 MRSA isolates, SCC*mec* II (39/53, 73.6%) was the most frequent SCC*mec* type as presented in Table 4, followed by SCC*mec* V (8/53, 15.1%), SCC*mec* IV (4/53, 7.5%) and SCC*mec* I (2/53, 3.8%). As shown in Fig. 1, produced by eBURST based on the ST data in this study, CC5 (ST5, ST764, ST965 and ST3066; 44/120, 36.7%) was the most common clonal complex (CC), followed by the livestock-associated (LA) clone CC398 (ST398, 14/120, 11.7%). Furthermore, MRSA-CC5 has been the dominant CC among MRSA isolates in this study and the percentage was as high as 69.8% (37/53), and all *tst*-positive MRSA isolates (n = 29) were CC5-MRSA as well. The *agrI* (66/120, 55%) was the most frequent *agr* group, followed by *agrII* (50/120, 41.7%), *agrIII* (2/120, 1.7%), and *agrIV* (2/120, 1.7%).

**Table 3:** Molecular characteristics of *Staphylococcus aureus* isolates causing bloodstream infections from 2013 to 2018.

ST	Isolates,n	MSSA, n	MRSA SCC*mec* Type(n)	*spa* type(n)	Virulence factors(n)
5	28	3	II (24) IV (1)	t1818 (1),t2460 (9),t264 (2),t311 (3),t450 (1),t458 (1), t601 (1), t9353 (2), t9363 (4) t2460 (1) t450 (1), t179 (1), t548 (1)	*tst*(21),*sea*(4),*seb*(1),*sec*(21),*seg*(24),*seh*(15),*sei*(22) *tst*(1), *sec*(1), *seg*(1), *sei*(1) *sed*(3), *seg*(3), *seh*(1),*sei*(3), *sej*(2)
764	12	2	II (8) IV (2)	t002 (8) t002 (2) t002 (2)	*tst*(3),*seb*(2),*sec*(3), *seg*(8),*seh*(8),*sei*(8) *tst*(2), *sec*(2), *seg*(2), *seh*(2), *sei*(2) *sec*(1), *sed*(1), *seg*(2), *seh*(1), *sei*(2), *sej*(1)
965	2	2		t062 (2)	*sea*(1),*seg*(2),*seh*(2),*sei*(2)
3066	2	0	II (2)	t5076 (2)	*tst*(2),*sec*(2),*seg*(2),*sei*(2)
398	14	10	I (1) V (3)	t034 (1) t034 (3) t571 (4),t034 (3),t1451 (1),t1184 (1), t18609 (1)	None *seh*(1) *seh*(3)
7	8	8		t796 (4), t091 (3), t605 (1)	*seh*(5)
199	6	6		t084 (2),t2325 (1),t279 (1),t346 (1),t803 (1)	*seh*(2)
1801	6	0	I (1) II (4) V (1)	t037 (1) t030 (1),t037 (2),t421 (1) t459 (1)	*sea*(1) *sea*(4), *seh*(2) *sea*(1)
1921	6	6		t164 (6)	*sei*(6),*seh*(3),*seg*(6)
6	5	5		t9121 (1),t18586 (1),t1131 (1), t304 (1), t701 (1)	*sea*(5),*sed*(1),*seh*(1),*eta*(1)
188	5	5		t189 (4),t4209 (1)	*tst*(1),*sec*(1),*seh*(4)
59	4	2	II (1) IV (1)	t437 (1) t437 (1) t437 (1),t441 (1)	*sea*(1) *seb*(1),*seh*(1) *sed*(1)
1821	3	2	V (1)	t4549 (1) t4549 (2)	None None
2959	2	2		t9353 (1),NT(1)	*seg*(2),*sei*(1)
1	2	2		t127 (2)	*sea*(1),*sec*(1),*seh*(1)
217	2	0	V (2)	t309 (2)	*pvl*(1), *sei*(2),*seg*(1)
630	1	0	V (1)	t2196 (1)	None
946	1	1		t437 (1)	*seg*(1),*sei*(1)
2315	1	1		t11687 (1)	*sec*(1),*seg*(1),*sei*(1)
2872	1	1		t6608 (1)	*seh*(1)
1281	1	1		t3277 (1)	*seg*(1),*seh*(1),*sei*(1)
72	1	1		t148 (1)	*tst*(1),*seg*(1)
683	1	1		t148 (1)	*sed*(1),*sej*(1)
45	1	1		t004 (1)	*seg*(1),*sei*(1)
1659	1	1		t774 (1)	*sed*(1)
25	1	1		t081 (1)	*seb*(1),*seg*(1),*sei*(1)
182	1	1		t616 (1)	*tst*(1),*seg*(1),*seh*(1),*sei*(1)
2867	1	1		t18585 (1)	None
858	1	1		t18607 (1)	None

ST, sequence type by multi-locus sequence typing; SCC*mec*, Staphylococcal cassette chromosome *mec*; *spa*, Staphylococcus protein A gene;

NT, not-typeable; None, no virulence gene detected.

**Table 4:** SCC*mec* types of 53 MRSA isolates causing bloodstream infections from 2013 to 2018.

SCC*mec* type(n)	ST(n)	*spa* type(n)	virulence factors(n)
I (2)	ST398 (1)	t034 (1)	None
	ST1801 (1)	t037 (1)	*sea*(1)
II (39)	ST5 (24)	t1818 (1),t2460 (9),t264 (2),t311 (3),t450 (1),t458 (1),t601 (1), t9353 (2),t9363 (4)	*tst*(21),*sea*(4),*seb*(1),*sec*(21),*seg*(24),*seh*(15),*sei*(22)
	ST764 (8)	t002 (8)	*tst*(3),*seb*(2),*sec*(3),*seg*(8),*seh*(8),*sei*(8)
	ST1801 (4)	t030 (1),t037 (2),t421 (1)	*sea*(4),*seh*(2)
	ST3066 (2)	t5076 (2)	*tst*(2),*sec*(2),*seg*(2),*sei*(2)
	ST59 (1)	t437 (1)	*sea*(1)
IV (4)	ST764 (2)	t002 (2)	*tst*(2),*sec*(2),*seg*(2),*seh*(2),*sei*(2)
	ST5 (1)	t2460 (1)	*tst*(1),*sec*(1),*seg*(1),*sei*(1)
	ST59 (1)	t437 (1)	*seb*(1),*seh*(1)
V (8)	ST398 (3)	t034 (3)	*seh*(1)
	ST217 (2)	t309 (2)	*pvl*(1),*seg*(1),*sei*(2)
	ST630 (1)	t2196 (1)	None
	ST1801 (1)	t459 (1)	*sea*(1)
	ST1821 (1)	t4549 (1)	None

SCC*mec*, Staphylococcal cassette chromosome *mec*; ST, sequence type by multi-locus sequence typing; *spa*, Staphylococcus protein A gene;

None, no virulence gene detected.

In 2013, the most common ST was ST5 (20.0%), followed by ST1801 (15.0%), ST7 (15.0%), ST764 (15.0%) and ST398 (10.0%); in 2014, the most common ST was ST5 (30.0%), followed by ST188 (15.0%), ST7 (15.0%) and ST764 (10.0%); in 2015, the most common ST was ST764 (25.0%), followed by ST1821 (15.0%), ST398 (10.0%), ST5 (10.0%) and ST199 (10.0%); in 2016, the most common ST was ST5 (40.0%), followed by ST398 (15.0%), ST59 (10.0%) and ST1921 (10.0%); in 2017, the most common ST was ST5 (20.0%), followed by ST1921 (10.0%); in 2018, the most common ST was ST398 (25.0%), followed by ST5 (20.0%). To sum up, ST5 (or CC5) has been highly prevalent among *S. aureus* isolates causing BSIs from 2013 to 2018 in Shanghai.

Eighty-one (67.5%) patients developed SAB 48 hours or more after admission to hospital that were considered as healthcare-associated (HA) infections, and 43 (53.1%) patients were infected with MRSA. ST5 (24/81, 29.6%) was the most common ST among HA-*S. aureus* isolates, and CC5 (36/81, 44.4%) was the most common CC as well. In addition, CC5-II MRSA (28/43, 65.1%) was the most frequent clone among HA-MRSA isolates.

## Discussion

*S. aureus* or MRSA is an important pathogen and frequent cause of invasive infections as well as bloodstream infections around the world. In this study, SAB accounted for 7.4% of BSIs cases in Shanghai from 2013 to 2018, much lower than that in a regional burn center in Jiangxi province in China, which is 25.9% from 2012 to 2016^8^. Coincidentally, in the United States, the incidence of MRSA BSIs in hospitals and communities dropped off 74% and 40% respectively from 2005 to 2016^17^. According to the latest data provided by the European Antimicrobial Resistance Surveillance Network (EARSNet) (2013–2016), more than a third of countries with low and high MRSA prevalence have reported significantly decreasing trends among bloodstream infections, and the population-weighted average MRSA BSIs percentage has dropped from 18.1% in 2013 to 13.7% in 2016^18^. The exact reasons for this specific decline among MRSA BSIs are not fully understood. However, in despite of these positive developments, *S. aureus* or MRSA still remains a priority for public health in Europe, with 10 of 30 countries reporting prevalence rates of MRSA > 25%, including Greece^19^.

The mortality rate of *S. aureus* BSIs in Shanghai was 25.8% from 2013 to 2018 in this study, similar to that reported in other published researches of adult patient with *S. aureus* BSIs (around 20–30%) in other countries^3,5,20^. However, it was much higher than that among infants with *S. aureus* BSIs both in Europe and USA which have shown overall mortality of 6.4–16%^7,21,22^. The prevalence of methicillin resistance among *S. aureus* isolates in Hong Kong has risen to >50% while the mortality rates of MRSA bloodstream infections are close to one-third as reported^23^. The proportion of MRSA among *S. aureus* BSIs in Shanghai was 44.2% in this study. Nevertheless, methicillin resistance is always significantly associated with higher mortality as well as comorbid conditions, intensive care unit admission, and prior exposure to antibiotics^24^. In addition, chronic lung disease, previous hospitalization and older patients (>79 years) are related with increased mortality as well^23^. The mortality in MSSA infections significantly declined and the average time to anti-staphylococcal therapy in MSSA infections decreased even though the mortality in MRSA infections was unchanged^25^. The declining mortality in MSSA infections might be related to the reduction in the duration of targeted therapy. These results emphasize the potential for rapid diagnostics and early optimization of treatment to impact outcomes in MSSA bacteremia. Nevertheless, it was revealed that the treatment failure rate of complicated MRSA bloodstream infections was as high as 40%^26^. MRSA might still need more attention when the patient was determined with MRSA bloodstream infections.

CC5 (*S. aureus*: 44/120, 36.7%; MRSA: 37/53, 69.8%) was the dominant clone among patients with SAB at Ruijin Hospital in Shanghai from 2013 to 2018 in this study, slightly different from our previous research conducted in Shanghai from 2009 to 2011 that CC8 (ST239) might be more common among patient with SAB before^27^. However, shifts may be occurring in the molecular epidemiology of *S. aureus* and MRSA among patients with bloodstream infections. As reported from a single-center surveillance in Greece, the widespread HA-MRSA clone ST239-III gradually decreased with the increase of the isolation frequency of these two CCs: HA-MRSA CC5, mostly belonging to ST5-II; and community-associated MRSA (CA-MRSA) CC80, mainly represented by ST80-IV-t044^19^. In this study, CC5- II MRSA was found in 34 isolates accounting for 91.9% (34/37) among CC5 MRSA isolates, suggesting that CC5 MRSA or CC5-II MRSA might be more frequent occurring among patients with MRSA BSIs in Shanghai. It has been suggested that CC5 has become an increasingly frequent cause of MRSA infections as well as bacteremia acquired by hospitalized patients from some other reports and that healthcare exposure is no longer a discriminator of USA100 vs USA300 infections^28–30^. An analysis of SAB conducted in Spain over 15 years (2002–2017) revealed that CC5 was the most prevalent CC and the proportion of CC5 among healthcare-associated *S. aureus* isolates was higher than that among community-associated *S. aureus* isolates, and CC5 was much more associated with methicillin resistance^4^. Simultaneously, CC5 MRSA was also prevalent in bloodstream infections in nine Latin American countries except in Colombia and Ecuador^29^. Clonal substitution appears to be a common phenomenon, and continuous surveillance is essential to identify molecular epidemiological changes in *S. aureus* and MRSA.

The livestock-associated clone CC398 was observed secondly frequent in this study. In our previous study, CC398 was the most common clone among patients with skin and soft tissue infections (SSTIs) especially where the livestock husbandry well developed in China^31^. In recent years in Denmark, the percentage of LA-MRSA CC398 among BSIs and SSTIs has been increasing and reached its peak in 2014, accounting for 16% and 21% in MRSA BSIs and SSTIs respectively^32^. The vast majority of patients with LA-MRSA CC398 BSIs had no history of exposure to livestock, which is similar to the ratio observed for other types of MRSA, and it demonstrates that the increase of LA-MRSA CC398 BSIs was accompanied by a large increase of LA-MRSA CC398 SSTIs and the expansion of porcine reservoir^32^. CC398 *S. aureus* resulted in an increasing incidence of bloodstream infections in a French hospital between 2010 and 2017 as well; the prevalence rate of CC398 isolates among *S. aureus* BSIs increased from 3.6% in 2010 to 20.2%in 2017 (*P* < 0.05)^33^. CC398 MRSA emerged but remains very sparse and CC398 MSSA disseminates in the community as suggested. In this study in Shanghai, 4 CC398 MRSA and 10 CC398 MSSA isolates were discovered. More recently, CC398 MSSA have been increasingly being reported as the cause of invasive infections among patients who have no contact with livestock, and CC398 MSSA bloodstream infections were always associated with high mortality^34^. It has been hypothesized that lysogeny may play an important role in increasing the ability of ST398 clone to cause human infections, and the significant risk calling for urgent attention is that ST398 clone family will still increase its threat to public health by continuing to obtain virulence and/or multidrug resistance genes from healthcare-associated *S. aureus* clones^35^. Therefore, it is deeply needed to monitor the extraordinary cloning of human adaptive *S. aureus* like CC398 and genomic studies might can figure out the determinants of its diffusion.

## Supplementary information

Supplementary information.
